# Left-Sided Upper Partial Anomalous Pulmonary Venous Return through a Curved Vein Joining the Left Brachiocephalic Vein

**DOI:** 10.1155/2016/1780909

**Published:** 2016-10-09

**Authors:** Eleonora Tricarico, Francesco Tricarico, Carlo Florio

**Affiliations:** ^1^Department of Radiology, Policlinico di Bari, Bari, Italy; ^2^Department of Radiology, P.O. San Paolo, Bari, Italy; ^3^Department of Radiology, C.B.H. Mater Dei, Bari, Italy

## Abstract

The evaluation of pulmonary veins during cross-sectional imaging of the chest and the knowledge of their embryology and anatomy are useful for detecting congenital conditions that may be clinically significant. Moreover, with the spread of cross-sectional imaging it is very frequent to find anatomical variants; therefore the radiologist should easily recognize their appearances. This case report shows a left-side upper partial anomalous pulmonary venous return (PAPVR) through a “curved” vein that joins the left brachiocephalic vein, in a female patient who underwent whole-body computed tomography (CT) for staging endometrial cancer. This was an incidental finding, not related to any symptoms; however, we explain the anatomical aspects of this abnormality within the congenital condition of PAPVR and its possible clinical relevance.

## 1. Introduction

The diffusion of cross-sectional imaging makes the detection of anatomical variants very frequent. PAPVR is an uncommon abnormality that may be incidentally detected at CT scans performed for other unrelated conditions, and it consists of one or more (but not all) anomalous pulmonary veins draining into a systemic vein [1, 2].

Our case presents a left-sided upper PAPVR through a “curved” vein that drains to left brachiocephalic vein. Another finding is an independent lingular vein that joins the left atrium.

Although PAPVR is often asymptomatic, its recognition may be important because the variability of pulmonary vein drainage to the left atrium may lead to a left-to-right shunt, can be significant in affecting the success of catheter ablation in patients with atrial fibrillation, and should be considered in the preoperative evaluation of patients with lung cancer [3].

To our knowledge, this is the first case that described a left-sided upper PAPVR through a “curved” vein, which goes first inferiorly and then medially to the left pulmonary artery to join the left brachiocephalic vein, because the cases of left-sided upper PAPVR reported in the literature typically present a “vertical” vein located laterally to the left pulmonary artery.

## 2. Case Report 

A 52-year-old woman undergoes whole-body CT for staging endometrial cancer. The patient does not present with any symptoms and her periodic cardiac evaluations for essential hypertension were unremarkable.

**Figure 1:**
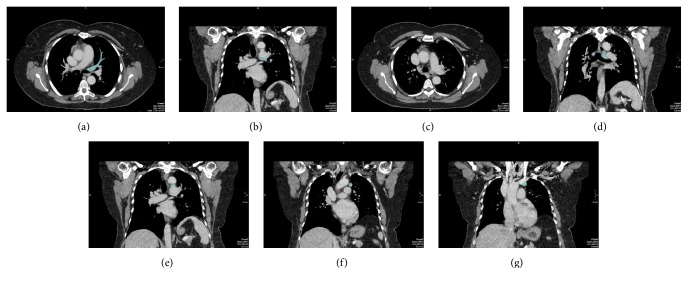
Axial and coronal sequential images: (a, b) the left upper pulmonary vein (light blue) first runs under the left pulmonary artery, (c, d) then goes up medially to it, (e, f) crosses the aortopulmonary window, and finally (g) joins the left brachiocephalic vein.

**Figure 2:**
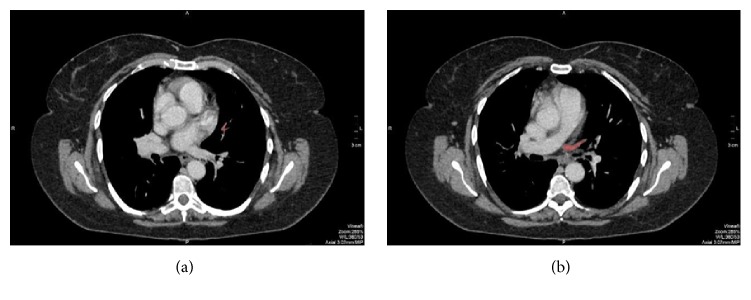
Axial sequential images: (a, b) the accessory lingular vein (red) presents orthotopic drainage to the left atrium.

**Figure 3:**
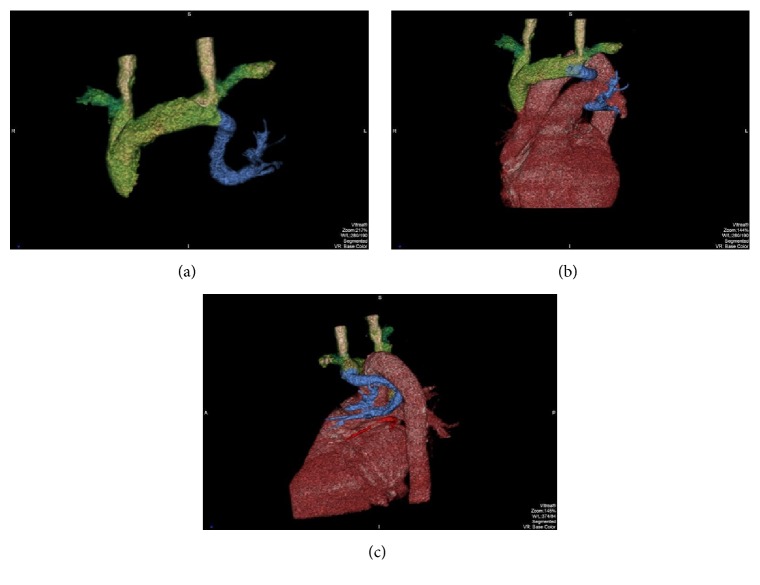
3D reconstructions: (a, b) the left upper pulmonary vein (light blue) describes a curved line with superior concavity, finally joining the left brachiocephalic vein; (c) the lingular vein (red) has independent drainage to the left atrium.

Contrast enhancement chest CT, performed in the portal venous phase as our usual staging protocol, reveals that the left upper pulmonary vein presents an anomalous route: it first runs under the left pulmonary artery, then goes up medially to it, runs via the aortopulmonary window, and finally joins the left brachiocephalic vein, describing on the whole a curved line with superior concavity (Figures 1 and 3). Another finding was an accessory lingular vein (Figures 2 and 3).

## 3. Discussion 

Partial anomalous pulmonary venous return (PAPVR) is a rare congenital abnormality in which one or more (but not all) of the pulmonary veins drain to the right atrium, directly or most frequently through one of its tributary veins [4]. The prevalence of PAPVR is about 0,4–0,7% and it is usually incidentally detected on cross-sectional imaging [2].

During the first two months of foetal development, the venous blood from the pulmonary vascular bed drains into systemic veins. After that common pulmonary vein arises from the left atrium and grows toward the lung, it develops a connection with the pulmonary vascular bed, with disappearing of the primitive connections with systemic veins. PAPVR arises from the failure of one or more of the pulmonary veins' connection with the common pulmonary vein and with the lack of the regression of part of primitive lung drainage to systemic vein [5].

Many authors suggest that right-sided PAPVR occurs twice as often as left-sided PAPVRs. However, other studies report that left-sided PAPVR may often be found in adult patients; instead right-sided PAPVR is more frequent in children [1].

Right-sided PAPVR consists of anomalous drainage of a right pulmonary vein to superior vein cava, azygos vein, coronary sinus, or inferior vena cava. Right-sided PAPVR with anomalous vein of right upper lobe draining to superior vein cava is considered one of the most frequent types of right PAPVR and it could be associated with an atrial septal defect [6].

Left-sided PAPVR usually drains into the left brachiocephalic vein, often through a vertical vein. Sometimes left-sided PAPVR drains via a persistent left SVC into the coronary sinus, a pericardiophrenic vein or the hemiazygos vein [1, 7].

Although PAPVR normally drains into a vessel located in the proximity, that is, into right-sided vein for right lung pulmonary veins and into a left-sided vein for the left lung pulmonary veins, some rare cases of “cross mediastinal drainage” are reported [7].

In our patient another finding is an orthotopic lingular vein that drains independently to the left atrium. The majority of people have four pulmonary veins, two right and two left. Sometimes there is a single pulmonary vein on one side, and it occurs more frequently on the left side [5]. Occasionally there are accessory pulmonary veins, which are named on the basis of the pulmonary lobe that they drain, and present an independent atriopulmonary venous junction separate from the superior and inferior pulmonary veins, without functional significance. Accessory drainage occurs more frequently on the right side, involving the right middle lobe or superior segment of the right lower lobe, so the presence of an accessory lingular vein draining independently to the left atrium is described as a rare anatomical variant [3, 8]. However, we cannot consider it to be a very rare finding associated with PAPVR, because most of the cases of PAPVR reported in the literature do not clearly reveal if they are associated with any accessory pulmonary veins. Moreover, some authors suggest that the presence of one or more accessory veins could be associated with PAPVR, and particularly cases of left-sided PAPVR into left brachiocephalic vein concerning the superior pulmonary vein with the lingular drainage being orthotopic are described in the literature [7].

PAPVR may be suspected at echocardiography; however, the lack of adequate acoustic windows makes it difficult to detect through this diagnostic technique [5].

CT and magnetic resonance (MR) imaging represent diagnostic techniques of choice in diagnosis and characterization of congenital pulmonary vein anomalies and in particular of PAPVR.

CT is considered as a first-line technique in the diagnosis of PAPVR, providing a great spatial resolution and short imaging times with identification of the anomalous vein and its drainage and with possibility of detecting tracheobronchial and lung abnormalities eventually associated [5, 7]. Moreover, PAPVR is often incidentally found during CT exams performed for other unrelated conditions.

CT examination should be performed with injection of contrast medium, although some authors suggest that PAPVR may be detected in nonenhanced CT acquisition.

The parameters of injections are high iodine concentration and flow rate of 4-5 cc/s, with a bolus trigger placed in the ascending aorta; our patient underwent whole-body CT for staging of cancer, so we performed chest CT in a venous phase, as our oncologic protocol suggests, but the anomalous vein was easily detected, and the 3D reconstructions allow us to clearly show the curved route of the abnormal left upper vein. Some authors suggest that left-sided PAPVR is easier to recognize than right-sided PAPVR on CT images, because of the high proportion of left PAPVR draining into a dilated left innominate vein through a vertical vein located laterally to the aortic arch, a finding that is easily detectable [7]. Normally cardiac gating is not required for the evaluation of pulmonary venous structure [2].

On CT images PAPVR of the left upper lobe can be mistaken for a left persistent superior vein cave (SVC) because both conditions are characterized by the presence of a “vertically oriented” anomalous vein, located laterally to the aortic arch; the main difference between these abnormalities is that in left persistent SVC CT scans show two vessels anteriorly to the left main bronchus, while in the left-upper lobe PAPVR there are not any vessels in that location, or there is one with a small caliber [9]. The singularity of our case of left upper PAPVR is represented by the curved route of the anomalous vein, which determines the presence of a venous vessel with a normal diameter located anteriorly to the left main bronchus before its curved route, contrary to all of the cases of upper left-sided PAPVR reported in the literature.

Use of ionizing radiation and intravenous iodinated contrast material that could adversely affect renal function represents the main disadvantage of CT [2]. Therefore, generally young patients with suspected PAPVR undergo MR.

MR imaging is an accurate technique in the evaluation of the anatomy of the pulmonary veins and may also demonstrate the presence of an associated atrial sept defect or other cardiac abnormalities [10]. There are many MR techniques used in the evaluation of the pulmonary venous system. Both black blood and bright blood images are acquired without IV injection of contrast medium. However, gadolinium-enhanced MR angiography is more accurate in defining the number and the type of the drainage of involved veins in PAPVR, because these images have a large field of view, present an excellent spatial resolution, and permit three-dimensional reconstruction. Moreover, gadolinium-enhanced MR angiography allows us to quantify the left-to-right shunt and right heart size with flow analysis and volumetry. This recognition of functional data could be important in the planning of the treatment of borderlines cases, because the right heart dilatation and the hemodynamically significant shunt represent indications for intervention [5].

The main disadvantages of MR are the protracted length of the examination, frequent need for patient sedation, eventual contraindications (i.e., pacemakers and defibrillators), and the scarce possibility of evaluation of eventual associated lung abnormalities [2, 7].

Patients with PAPVR are usually asymptomatic or may present with heart murmurs, fatigue, dyspnea, and arrhythmias. Some authors suggested that PAPVR may be clinically significant when 50% or more of the pulmonary blood drains to systemic circulation, resulting in a significant left-to-right shunt [2, 5]. This left-to-right shunt, if significant, causes an increase in pulmonary blood flow leading to progressive remodelling of the pulmonary circulation with increased pulmonary vascular resistance and development of pulmonary hypertension. In this cases, the heart volume overload causes, over the years, gradual right heart remodelling and sometimes severe tricuspid regurgitation and right atrial arrhythmias that may lead to sudden cardiac decompensation. Therefore, the early imaging recognition of PAPVR is important in preventing pulmonary hypertension and its consequences [11]; moreover, the knowledge of CT findings typical of PAPVR is essential to differentiate this congenital situation from other causes of pulmonary hypertension [12].

Anatomy of pulmonary veins is also important in the planning of percutaneous atrial fibrillation ablation, because the variability of pulmonary vein morphology may affect the success of this procedure, particularly if the presence of anatomic variant is not considered. In fact, the presence of pulmonary veins' anatomic variations leads to different ways of procedure [3].

Imaging detection of pulmonary veins' anatomic variant and particularly of PAPVR is important in patients that are candidates for lobectomy for a primary lung neoplasm; in case of PAPVR that involves a pulmonary lobe other than that affected by the cancer, a possible right-sided heart failure may occur, because of increased blood flow through this anomalous vein. Therefore, in those patients, PAPVR should be corrected during lobectomy. This situation does not occur if the neoplasm involves the pulmonary lobe with PAPVR, because the anomalous vessel is resected with lobectomy [1, 13].

In asymptomatic patients without significant shunt and signs of right heart overload surgery is not necessary. Patients with PAPVR presenting with right ventricle enlargement, tricuspid regurgitation, or clinical symptoms should undergo surgical repair to avoid the development of pulmonary hypertension; surgery consists in implanting the anomalous vein directly into the left auricular appendage or the left atrium [14, 15]. In the case of advanced pulmonary hypertension, the transplant is the only option [4].

Our patient's cardiological evaluation did not reveal any abnormalities, so she continued with periodic follow-up without any treatments related to PAPVR.

## 4. Conclusion 

PAPVR is a rare anatomic variant and the radiologist should have familiarity with its appearance, because it may be significant in some clinical situations. The distinctive tract of our case in the setting of left-sided upper PAPVR is the curved route of the anomalous vein, that, to our knowledge, has never been described in the literature.
